# Lonely at Work: The Moderating Role of Leader–Member Exchange in the Association Between Workplace Loneliness and Perceived Task and Contextual Performance

**DOI:** 10.3390/bs16091608

**Published:** 2026-09-09

**Authors:** Saadet Nur Karadenizli Sinap, Pinar Bayhan Karapinar, Ozge Tayfur Ekmekci

**Affiliations:** Department of Business Administration, Hacettepe University, Beytepe, 06800 Ankara, Türkiye; pbayhan@hacettepe.edu.tr (P.B.K.); otayfur@hacettepe.edu.tr (O.T.E.)

**Keywords:** workplace loneliness, leader–member exchange, task performance, contextual performance, employee performance, subordinate–manager relationship

## Abstract

The current study examined the moderating role of leader–member exchange (LMX) in the associations between workplace loneliness and employees’ perceived task and perceived contextual performance. Specifically, the study explored whether the negative associations between workplace loneliness and these two dimensions of performance were weaker at higher levels of LMX. Data were collected through a questionnaire-based survey of 352 white-collar employees from various sectors in Türkiye, and the hypotheses were tested using moderation analysis. Workplace loneliness was negatively associated with both perceived task and perceived contextual performance, in line with Hypotheses 1 and 2. Contrary to expectations, high-quality exchange relationships between subordinates and managers strengthened, rather than weakened, these negative associations for both perceived task and perceived contextual performance; H3 and H4 were therefore not supported. This pattern, which was not hypothesized a priori, points to a potential dark side of high-quality LMX and identifies LMX as an unanticipated boundary condition in the relationships between workplace loneliness and perceived performance. This pattern may be tentatively interpreted through a betrayal framework, whereby unmet relational expectations in high-quality LMX relationships may help account for the stronger negative association between loneliness and perceived performance. These findings extend the workplace loneliness and LMX studies by offering a possible betrayal-based interpretation of how relational quality may shape the loneliness–performance link and highlight the need for organizations to consider employees’ broader workplace relationships in addition to the quality of their relationships with supervisors.

## 1. Introduction

Loneliness has been listed as a public health concern, becoming a global pandemic that affects individuals of all ages, socio-economic backgrounds, and genders (World Health Organization, n.d.). Previous studies reported a connection between loneliness and various adverse health outcomes, including depression, faster cognitive decline, compromised cardiovascular health, and weakened immune responses (Ost-Mor et al., 2023; Vismara et al., 2022). Despite the prevalence of loneliness and its documented consequences, studies specifically examining workplace loneliness remain sparse (Anand & Mishra, 2021; Firoz & Chaudhary, 2022; Jin & Ikeda, 2024). This gap is particularly concerning, as loneliness has increasingly emerged as a stressor for employees, many of whom lack close friendships in their work environment. In fact, a recent study by Gallup (Gallup, 2024) found that 20% of participants reported having no close friends at work. In this sense, workplace loneliness could be conceptualized as “*employees’ subjective affective evaluations of, and feelings about, whether their affiliation needs are being met by the people they work with and the organizations they work for*” (Ozcelik & Barsade, 2018, p. 2345). Put differently, workplace loneliness reflects a perceived deficiency in the quality of interpersonal relationships and social interactions at work (Wright et al., 2006).

A growing body of research links workplace loneliness to a range of adverse employee attitudes and behaviors. To date, most research has focused on workplace loneliness as a persistent and enduring experience. When employees experience loneliness in a work environment, its negative behavioral and attitudinal consequences are likely to be particularly pronounced (McCarthy et al., 2026). Consistent with this view, workplace loneliness has been associated with lower organizational citizenship behavior (Firoz & Chaudhary, 2022; Lam & Lau, 2012), job satisfaction (Basit & Nauman, 2023), and well-being (Mohapatra et al., 2023), as well as higher work-family conflict (Firoz & Chaudhary, 2022) and work alienation (Mohapatra et al., 2023).

Albeit not the sole one, impaired job performance has also emerged as one of the most prominent adverse outcomes of workplace loneliness (Amarat et al., 2019; Ozcelik & Barsade, 2018). This study examines the associations between workplace loneliness and employees’ perceived performance, conceptualized along two related yet distinct dimensions: task and contextual performance. Task performance refers to “the effectiveness with which job incumbents perform activities that contribute to the organization’s technical core either directly by implementing a part of its technological process, or indirectly by providing it with needed materials or services” (Borman & Motowidlo, 1993), while contextual performance refers to “behaviors that contribute to shaping and maintaining the wider organizational and social context” (Motowidlo et al., 2014). Although empirically correlated, these dimensions are conceptually distinct and independently contribute to overall performance, being influenced by both shared and distinct antecedents, such as aptitude, training, personality, and experience, to varying degrees (Motowidlo et al., 2014). Given that workplace loneliness may be differentially associated with these dimensions, it is important to examine its relationship with each separately. The proposed relationships are grounded in the Conservation of Resources theory (Hobfoll, 1989), which posits that individuals are motivated to acquire, protect, and invest resources that are personally valuable. Within this framework, workplace loneliness may represent a state of social resource deprivation, threatening the relational and emotional resources employees depend on to sustain their performance (cf. Lee & Hong, 2025).

Yet not all employees experiencing workplace loneliness report equally negative outcomes, suggesting the presence of boundary conditions that shape this relationship. Building on this assertion, Firoz and Chaudhary (2022) and Xin et al. (2024) have emphasized the need for a deeper examination of factors that can reduce the adverse effects of loneliness. In response to these calls, the current study examines the boundary conditions that may shape the association between workplace loneliness and perceived performance. Given their organizational position, leaders serve as a powerful source of both emotional and material resources for employees, with the capacity to shape employees’ emotional states and resource availability both directly and indirectly (Hu & Shi, 2015; Wright et al., 2006; Yaffe & Kark, 2011; Zhou et al., 2023). In this context, some studies have examined how leadership behaviors relate to workplace loneliness and whether these leadership behaviors mitigate the adverse impacts of employees’ perceived loneliness (Hou & Cai, 2024; Nie et al., 2023; Xin et al., 2024; H. Yang et al., 2023).

The LMX theory examines various leadership behaviors and the quality of interactions between leaders and employees, which can lead to either low- or high-quality exchanges (Hou & Cai, 2024). Drawing on Conservation of Resources (COR) Theory (Hobfoll, 1989), this study examines the interaction between workplace loneliness and LMX in relation to perceived task and perceived contextual performance. This study contributes to the extant literature in several ways. First, by answering recent calls (McCarthy et al., 2026) to adopt a COR perspective on loneliness, we conceptualize LMX as a valuable relational resource that may shape the resource-draining effects of workplace loneliness on performance, thereby offering a resource-based explanation of how supervisory relationship quality may be associated with the performance implications of workplace loneliness. Second, this study integrates two relational domains that have generally been examined separately and offers a more nuanced understanding of how different workplace relationships are jointly associated with employees’ perceptions of their performance. In this vein, the study simultaneously addressed both workplace loneliness, which mostly captures interpersonal relations with co-workers, and LMX, which pertains to the relationship between leaders and employees. Third, by examining perceived task and perceived contextual performance separately, the study further clarifies whether this relational interplay extends across both core-role and socially embedded dimensions of performance.

## 2. Theoretical Framework

### 2.1. Workplace Loneliness and Perceived Performance: Task and Contextual Dimensions

Workplace loneliness arises as a result of the lack of the desired level of support, opportunity, and communication within the organization (Wright et al., 2006). To date, studies conducted with data from different industries and countries indicate that employees who suffer from loneliness at work tend to perform poorly across various performance indicators (Akçit & Barutçu, 2017; Lam & Lau, 2012; Ozcelik & Barsade, 2018). For instance, nurses reporting emotional and social loneliness were found to have lower work performance (Amarat et al., 2019). Similarly, data collected from Indian employees in manufacturing and service sectors revealed that lonesome employees had lower creative performance compared to employees not feeling loneliness (Firoz & Chaudhary, 2022). Using the data from forty-nine studies, a recent meta-analysis reported a medium to strong level of correlation between workplace loneliness and job performance (Bryan et al., 2023).

Loneliness in the workplace may be negatively associated with task performance through reduced social connections and communication with other members of the organization (Amarat et al., 2019; Lam & Lau, 2012). Lonely individuals could be more doubtful about the intentions of others, which obstructs social interactions in the workplace. In the work setting, lonely individuals might be less inclined to engage in social interactions with colleagues due to their apprehension that their contributions will not be acknowledged (Lam & Lau, 2012). Because they are deprived of interpersonal connections, lonely employees may be unable to obtain sufficient information (Bryan et al., 2023) and may be less willing to exert effort to satisfy their leaders (Chen et al., 2016; Peng et al., 2017). Workplace loneliness may also be associated with less favorable evaluations of employees’ own abilities, and thus, these employees tend to be less willing to accept and perform the tasks (Lam & Lau, 2012).

Workplace loneliness could be examined through the lens of Social Exchange Theory (Blau, 1964) or Belongingness Theory (Baumeister & Leary, 1995), which emphasize reciprocal obligations and the fundamental need to belong, respectively. However, COR theory (Hobfoll, 1989) offers a more direct account of the performance consequences examined here, as it explicitly links resource depletion to behavioral outcomes rather than focusing primarily on relational exchange norms or affiliation needs per se. COR posits that individuals strive to acquire, retain, and protect resources—including social, emotional, and informational resources—and that resource loss or the threat of loss constitutes the primary driver of stress and performance impairment. Workplace loneliness inherently represents a state of social resource deprivation reflecting a deficit of peer connections, informational exchanges, and sense of belonging that are central to employees’ effective functioning within organizational life (Lee & Hong, 2025). These resource deficits deplete the motivational and cognitive reserves necessary to sustain task performance. Moreover, COR’s loss spiral principle suggests that initial resource loss (i.e., felt loneliness) makes individuals more vulnerable to subsequent losses, compounding the performance costs over time. Beyond resource depletion, recent diary research further demonstrated that transient workplace loneliness negatively affects employees’ perceived task performance through both affective pathways, such as work frustration, and cognitive pathways, such as decreased work engagement, highlighting the multifaceted mechanisms underlying this relationship (Li et al., 2026). In sum, workplace loneliness emerges as a multifaceted threat to job performance, operating through both chronic resource erosion and affective and cognitive disruptions. Therefore, we propose:

**H1.**  *Workplace loneliness is negatively associated with perceived task performance.*

The resource-depleting effects of workplace loneliness, as described above (H1), may also undermine contextual performance, which refers to behaviors that extend beyond formal role requirements to support the broader social and organizational fabric of the workplace (Motowidlo et al., 2014). However, contextual performance is likely to be even more susceptible to this depletion than task performance, as it is inherently discretionary. In contrast to task performance, which is associated with formal role obligations, contextual performance requires employees to voluntarily allocate surplus resources including time, emotional energy, and a sense of belonging beyond the formal requirements of their position (Motowidlo et al., 2014). This discretionary surplus is likely the first to be withdrawn as workplace loneliness depletes the resources available for voluntary, extra-role contributions, prompting employees to redirect their remaining resources toward self-protection rather than organizational benefit (Ozcelik & Barsade, 2018). This reasoning is consistent with the fact that individuals who are experiencing workplace loneliness are more likely to believe that their organization is not meeting their requirements for social interaction and belonging, which further reduces their willingness to exceed formal expectations (Lam & Lau, 2012).

**H2.**  *Workplace loneliness is negatively associated with perceived contextual performance.*

### 2.2. LMX as a Moderator of the Associations Between Workplace Loneliness and Perceived Job Performance

LMX is a dyadic exchange relationship that focuses on the quality of dyadic interaction between an employee and their supervisor (Hou & Cai, 2024). The main premise of the LMX relationship is that leaders develop varying levels of exchange relationships with employees, ranging from low to high quality, which significantly influence employees’ attitudes and behaviors in the workplace (Çevik, 2025; Ilies et al., 2007; Ilsev et al., 2024). A substantial body of research documents the benefits of high-quality LMX, including enhanced job satisfaction, greater organizational commitment, and improved job performance (Dulebohn et al., 2012; Ferris et al., 2009; Sparrowe & Liden, 1997). High-quality LMX constitutes a valuable source of social and informational capital for employees. Supervisors in high-quality relationships share task-relevant knowledge, provide mentoring, assign challenging work, and offer emotional support (Bauer & Green, 1996; Breevaart et al., 2014; Chen et al., 2016; Law et al., 2000; Van Dam et al., 2008), thereby replenishing the resources that workplace loneliness erodes. When employees gain access to such resources through their supervisory relationship, they may be better equipped to offset the resource deficits imposed by felt loneliness, sustaining their task performance despite the adverse social conditions they experience at the peer level. On these grounds, we propose:

**H3.**  *LMX moderates the negative association between workplace loneliness and perceived task performance, such that this association is weaker when LMX quality is high.*

It should be noted that a growing body of research has also uncovered a “dark side” of high-quality LMX that complicates a straightforward resource-based account (Anand & Mishra, 2021; Wu, 2018). High-quality exchange relationships carry heightened relational obligations: employees who enjoy privileged access to their supervisor’s resources and attention may simultaneously experience intensified performance pressure, elevated expectations, and a heightened sensitivity to any imbalance in their broader workplace relationships (Martin et al., 2016; Wu, 2018). In addition, the betrayal framework (Restubog et al., 2010) suggests that employees who invest trust in a high-quality relationship may experience intense disappointment when that relationship fails to protect them from adverse work conditions, potentially leading to disengagement rather than resilience. Empirically, Anand and Mishra (2021) demonstrated that the positive relationship between loneliness and emotional exhaustion was stronger, not weaker, for employees with high-quality LMX. This literature raises the possibility that, under some conditions, high-quality LMX could intensify rather than buffer the adverse effects of workplace loneliness. However, because the loneliness examined here reflects a deficiency of relational resources at the peer level rather than a deficit of supervisory assistance per se and because high-quality LMX has more consistently been linked to superior employee outcomes in the broader meta-analytic literature (Dulebohn et al., 2012; Ferris et al., 2009; Sparrowe & Liden, 1997), we retained the resource-substitution logic above as our primary, a priori prediction (H3).

The same reasoning applies to contextual performance. Paralleling the task–contextual distinction established earlier (H1–H2), task performance is tied to formal role obligations and can therefore be sustained once a baseline resource deficit is compensated, whereas contextual performance is discretionary. It involves time, emotional energy, and motivation invested voluntarily beyond what the job formally demands and requires a resource surplus beyond this baseline. High-quality LMX may be particularly well suited to generating this surplus: beyond replenishing depleted resources, it offers employees clear signals of supervisor support, encouragement, and recognition (Breevaart et al., 2014), which can restore not only the resources lost to loneliness but also the motivational reserve required to voluntarily exceed one’s formal role (Jawahar & Carr, 2007; Swift & Virick, 2013; Wayne et al., 1997). We therefore propose:

**H4.**  *LMX moderates the negative association between workplace loneliness and perceived contextual performance, such that this association is weaker when LMX quality is high.*

**Figure 1:**
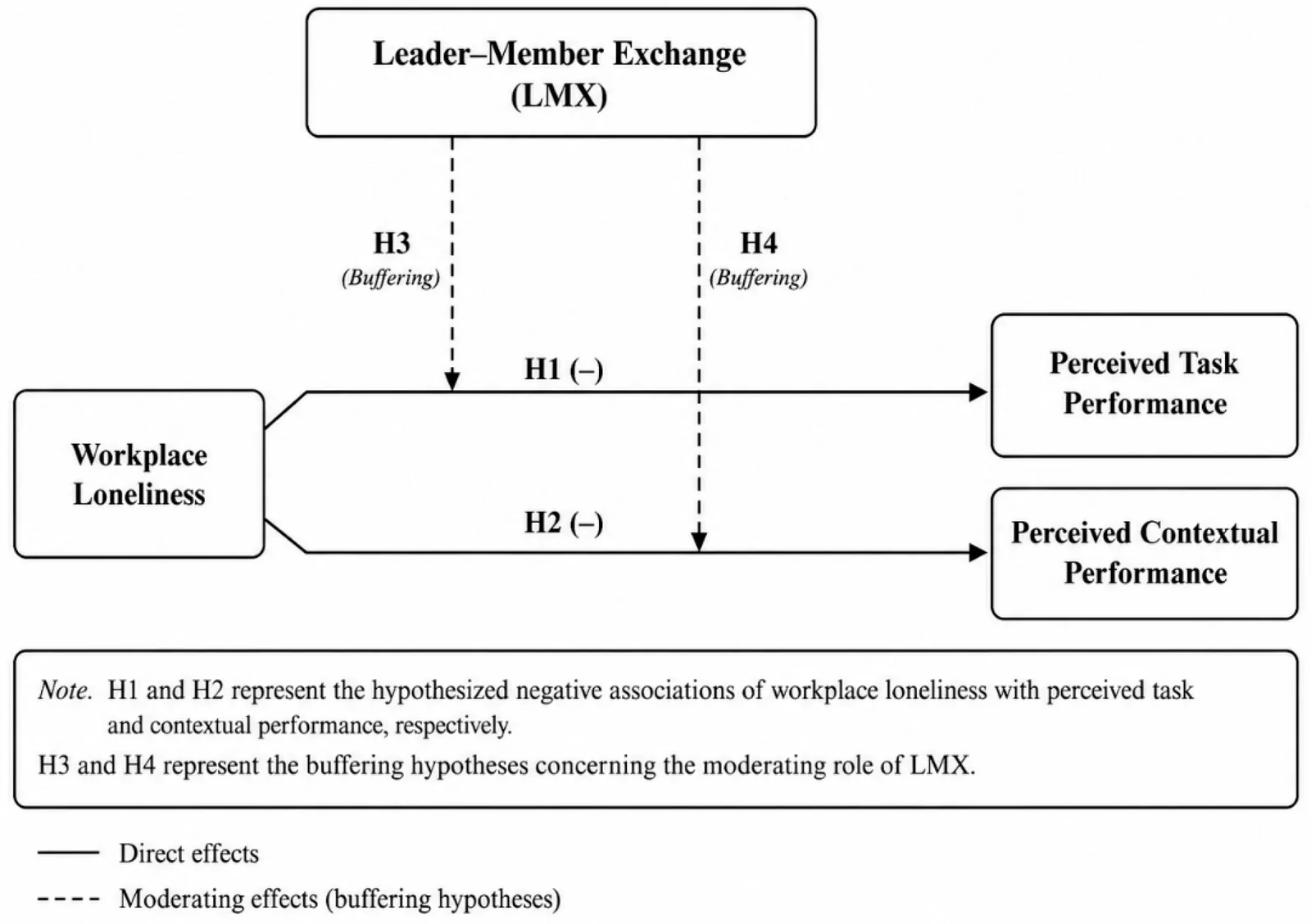
Conceptual Research Model.

The proposed relationships among workplace loneliness, LMX, and perceived task and perceived contextual performance are summarized in the conceptual research model presented in Figure 1.

## 3. Materials and Methods

### 3.1. Participants and Procedure

Some studies (Wright, 2005) have shown that employees with higher degrees or those who spend long hours in isolation experience higher levels of workplace loneliness. Following this corollary, it seems important to examine the association between workplace loneliness and job performance among white-collar employees, given that these employees often possess higher educational qualifications and frequently work alone in office settings. Therefore, it seems important to gather data from white-collar employees to understand the plausible role of LMX on loneliness–performance linkage. Hence, this study utilized data from Turkish professionals employed in both the public and private sectors. Turkish culture has been characterized as relatively collectivistic, with a preference for harmonious relationships with supervisors and co-workers (Kağıtçıbaşı, 1996). This cultural context provides a broader backdrop for considering how workplace relationships are perceived and experienced.

The present study was conducted subsequent to obtaining ethical approval. Before data collection, participants were informed that their participation was entirely voluntary and assured that all collected data would be kept confidential and anonymous. The study commenced with the selection of participants who met the sampling criteria, which included being employed in a white-collar profession and having a direct supervisor. Snowball sampling was used because access to white-collar employees who met the inclusion criteria and had a direct supervisor was facilitated through participants’ professional networks. The data collection process lasted six months, during which a total of 404 individuals participated in the study. During the data-screening process, 52 cases were identified as univariate outliers based on the |Z| > 3.29 criterion and were therefore excluded from the analyses. Consequently, the final analytic sample consisted of 352 participants. A comparison of the excluded and retained cases revealed no meaningful demographic differences between the two groups, suggesting that their exclusion is unlikely to have introduced systematic demographic bias into the final sample.

The adequacy of the sample size was checked on the basis of statistical power using an F-test approach for multiple regression with moderation (Cohen, 1988). For all models, the effect size f^2^ was computed using the observed R^2^ for the overall model (f^2^ = R^2^/(1 − R^2^)) and the t-statistic of the interaction term (f^2^ = t^2^/df) for the moderation effect. Power was then calculated from f^2^, the numerator and denominator degrees of freedom, and α = 0.05 using the noncentral F distribution. A sensitivity analysis further identified the smallest effect size that could be detected with 80% power at α = 0.05 for the present sample (*N* = 352). A post hoc power analysis revealed that power was nearly at ceiling level (power > 0.999) for the overall regression models for both the task performance model (f^2^ = 0.149, R^2^ = 0.13) and the contextual performance model (f^2^ = 0.266, R^2^ = 0.21). Thus, the sample size was sufficient to detect effects of this magnitude. For the interaction (moderation) terms, the obtained power was 0.986 for the contextual performance model (f^2^ = 0.050) and 0.770 for the task performance model (f^2^ = 0.021), with the latter falling slightly below the conventional 0.80 benchmark. Rather than treating this lower power as increasing confidence in the effect, we note that the task-performance interaction remained statistically significant (b = −0.11, SE = 0.04, *p* = 0.007, 95% CI [−0.20, −0.03]), though its point estimate carries somewhat greater sampling uncertainty than the corresponding contextual-performance interaction. This effect should therefore be interpreted alongside its effect size and confidence interval, together with the sensitivity-analysis results reported below, rather than on the basis of statistical significance alone. The sensitivity analysis further indicated that, with the current sample size, α = 0.05, and the present model specifications, the study had 80% power to detect effects as small as f^2^ ≈ 0.02–0.03 (Cohen, 1988), suggesting that the design was adequately powered to detect even small effects.

Table 1 presents the demographic characteristics of the sample. The majority of participants held a bachelor’s degree (68.2%) and worked in the public sector (63.6%), and the sample was fairly evenly split by gender (52.3% female, 47.7% male).

### 3.2. Measures

Participants were asked questions related to demographic information (i.e., gender, age, education level), as well as scale items pertaining to workplace loneliness, LMX, and dimensions of job performance. Participants indicated their agreement with the scale items using a 5-point Likert scale ranging from 1 (strongly disagree) to 5 (strongly agree). Higher scores indicated higher-quality LMX, greater workplace loneliness, and higher levels of perceived task and perceived contextual performance.

Leader–Member Exchange Scale: The seven-item scale developed by Graen and Uhl-Bien (1995) is used to assess the characteristics of the working relationships between managers and employees. The items are intended to measure the employees’ evaluations of their supervisors in terms of their professional capabilities and behaviors (Sample item: “*I have enough confidence in my supervisor/manager that I would defend and justify his or her decisions if he or she was not present to do so*”). Since the scale had already been translated into Turkish by Çalışkan (2015), there was no need for re-translation. In the initial study carried out by Graen and Uhl-Bien (1995), the Cronbach’s α coefficient was found to vary from 0.80 to 0.90. In the Turkish adaptation, Çalışkan (2015) obtained a Cronbach’s α of 0.84. The current research obtained an equivalent level of reliability (Cronbach’s alpha = 0.87). The leader–member exchange scale further demonstrated adequate psychometric properties, with a composite reliability (CR) value of 0.880 and an average variance extracted (AVE) value of 0.518. These results provide support for the reliability and convergent validity of the construct.

Workplace Loneliness Scale: This study employed the sixteen-item scale created by Wright et al. (2006), which was subsequently adapted into Turkish by Dogan et al. (2009). In accordance with the original scoring procedure, items designated for reverse scoring (Items 5, 6, 10, 11, 12, 14, 15, and 16) were reverse-coded before the analyses, ensuring that higher composite scores consistently reflected higher levels of workplace loneliness. The workplace loneliness scale is composed of two dimensions, encompassing emotional deprivation (Sample item: “*I often feel emotionally distant from the people I work with*”) and social companionship (Sample item: “*There is someone at work I can talk to about my day-to-day work problems if I need to*”). Emotional deprivation captures the perception of the emotional quality of one’s workplace relationships; lack of social companionship, on the other hand, refers to the perception of quantifiable social aspects of those relationships (Wright et al., 2006). Although earlier studies (e.g., Amarat et al., 2019) have treated workplace loneliness as a single, undifferentiated factor, the present study specified it as a second-order factor comprising emotional deprivation and social companionship as first-order dimensions, based on the dimensionality analysis reported in the Confirmatory Factor Analysis section below. Wright et al. (2006) reported the Cronbach’s alpha coefficient for the original scale as “0.80”; Dogan et al. (2009) reported the alpha value as “0.90” for the Turkish adaptation of the scale. The present study determined Cronbach’s alpha coefficient to be “0.93”. The workplace loneliness scale also showed strong psychometric adequacy, with a CR value of 0.922. The AVE value was 0.432, which was slightly below the commonly recommended 0.50 criterion. Considering the high CR value, the construct was regarded as demonstrating acceptable convergent adequacy, consistent with methodological recommendations suggesting that AVE values slightly below 0.50 may be acceptable when composite reliability is sufficiently high (Hair et al., 2014).

Job Performance Scales: The measurement of perceived task and perceived contextual performance was conducted using two distinct scales, both designed to evaluate employees’ self-assessments of their own performance. Accordingly, task and contextual performance in the present study refer to employees’ self-reported or perceived performance rather than supervisor-rated or objective performance (sample item: “Demonstrates the fulfillment of duties outlined in the job description”). Perceived task performance was measured using the in-role behavior subscale developed by Williams and Anderson (1991) and adapted into Turkish by Çam (2019). The original study by Williams and Anderson (1991) reported a Cronbach’s alpha coefficient of 0.91. According to Çam (2019), the Cronbach’s alpha coefficient obtained from the Turkish adaptation was found to be 0.88. In the current study, the Cronbach’s alpha coefficient was found to be “0.90”. The perceived task performance scale exhibited strong psychometric properties, with a CR value of 0.920 and an AVE value of 0.628. These findings indicate that the construct met the recommended standards for reliability and convergent validity.

Perceived contextual performance was measured using the 16-item scale developed by Motowidlo and Van Scotter (1994) to assess the contextual performance dimensions identified by Borman and Motowidlo (1993). The sample item is “*Voluntarily do more than the job requires to help*”. The Cronbach’s alpha coefficient for the original scale is reported as “0.95”. In the Turkish adaptation study (Çam, 2019), the scale’s Cronbach’s alpha was determined to be 0.90. The alpha coefficient was 0.90 for the current study. The contextual performance scale also demonstrated strong composite reliability, with a CR value of 0.913. Although the AVE value was 0.403, the high CR value indicates that the construct retained acceptable measurement reliability. In line with prior methodological guidance suggesting that AVE values below 0.50 may still be acceptable when accompanied by strong composite reliability, the construct was retained in the measurement model (Hair et al., 2014). Data screening and statistical analyses were conducted using IBM SPSS Statistics, version 23.0 (IBM Corp., Armonk, NY, USA), and confirmatory factor analyses were performed using IBM SPSS Amos, version 23.0 (IBM Corp., Armonk, NY, USA).

## 4. Results

### 4.1. Preliminary Analyses

Before the main analyses, the dataset was screened for data-entry errors, missing data, and outlying observations. Univariate outliers were examined using standardized Z scores, histograms, and boxplots, with |Z| > 3.29 used as the screening criterion (Tabachnick & Fidell, 2014). Multivariate outliers were assessed using Mahalanobis distance and calculated across all 46 items comprising the four focal constructs (LMX, workplace loneliness, task performance, and contextual performance), with degrees of freedom equal to the number of items entered (df = 46) and a chi-square critical value evaluated at *p* < 0.001 (χ^2^crit = 81.40). Nineteen cases in the retained sample exceeded this critical value. Inspection of these cases indicated that they did not differ significantly from the remaining sample on workplace loneliness, task performance, or contextual performance, but reported substantially lower LMX scores (M = 2.53 vs. M = 3.41 among retained cases), t(350) = 4.92, *p* < 0.001. Because LMX is a focal moderator in the present study, these 19 cases appear to reflect a theoretically meaningful subgroup of employees with markedly low-quality leader–member relationships rather than random measurement error or careless responding; excluding them would have artificially restricted the range of this key moderating variable and could have biased the estimated interaction effects. They were therefore retained in the final analytic sample. As a sensitivity check, the principal regression models were re-estimated after excluding these 19 cases (*N* = 333); the interaction effects retained the same direction and remained statistically significant for both contextual performance (b = −0.15, *p* < 0.001) and task performance (b = −0.10, *p* = 0.037), indicating that the reported findings are not an artifact of these particular cases. Following data screening, normality was examined using skewness and kurtosis values. Because all values fell within the range of −2 to +2 (George & Mallery, 2020), no severe violations of normality were detected. Potential multicollinearity was also examined using variance inflation factor (VIF) values. All VIF values were below 2.50, indicating that multicollinearity was not a substantial concern.

Because workplace loneliness has been conceptualized as comprising two distinct dimensions—emotional deprivation and social companionship (Wright et al., 2006)—we compared a one-factor model, a two-factor model with emotional deprivation and social companionship as correlated first-order factors, and a second-order model in which both first-order dimensions were loaded onto a general workplace loneliness factor. The one-factor model showed poor fit (χ^2^ (104) = 712.99, CFI = 0.822, RMSEA = 0.129). The two-factor model showed a significant improvement in fit (Δχ^2^ (1) = 229.88, *p* < 0.001; CFI = 0.889, RMSEA = 0.103), confirming that emotional deprivation and social companionship should be treated as distinct dimensions rather than collapsed into a single undifferentiated factor. The second-order model, in which a general workplace loneliness factor accounted for the covariation between the two first-order dimensions, produced statistically equivalent fit to the two-factor model (Δχ^2^(1) ≈ 0.00, *p* > 0.05) while yielding strong and significant second-order loadings (ED → WLON = 0.995; SC → WLON = 0.818, both *p* < 0.001). The second-order model was identified following standard practice for higher-order factor models. Specifically, the loading of the emotional deprivation (ED) first-order factor on the general workplace loneliness (WLON) factor was fixed to 1 to set the scale of WLON, whose variance was then freely estimated, while the loading of the social companionship (SC) first-order factor on WLON was freely estimated. With two first-order indicators, this second-order component of the model is just-identified in isolation; the overall model nonetheless remains over-identified because ED and SC are each themselves defined by multiple observed items. Given the strength of these second-order loadings and our theoretical interest in a unified workplace loneliness construct, we retained the second-order structure for subsequent analyses. We note that, with only two first-order factors, the second-order model is mathematically equivalent in fit to the correlated two-factor model (identical χ^2^, df, and all other fit indices), since fixing a single higher-order factor to explain the covariance between exactly two first-order factors imposes no additional constraint beyond allowing them to correlate. The two representations are therefore statistically indistinguishable, and our preference for the second-order specification was accordingly a theoretical rather than an empirical one: it directly operationalizes our conceptualization of workplace loneliness as a unified construct comprising emotional deprivation and social companionship as manifestations of a common underlying experience, consistent with Wright et al.’s (2006) original conceptualization and with how workplace loneliness is treated throughout our hypotheses (H1–H4), rather than treating the two dimensions as separate predictors.

After data screening, several confirmatory factor analyses (CFAs) were conducted to assess whether scale items were significantly associated with the study variables. A four-factor measurement model comprising LMX, workplace loneliness (specified as a second-order factor, with emotional deprivation and social companionship as first-order dimensions), task performance, and contextual performance and a three-factor model comprising LMX, workplace loneliness, and performance (performance dimensions combined) were specified. The nested model comparison indicated the superiority of the four-factor model over the three-factor model (Δχ^2^(3) = 476.30, *p* < 0.001), supporting the treatment of task and contextual performance as separate dimensions. However, the initial four-factor model did not fit the data well. Inspection of modification indices revealed several large residual covariances among items sharing highly similar wording within the same construct; specifically, 9 such pairs were identified within the workplace loneliness scale (IYY3–IYY4, IYY5–IYY6, IYY2–IYY9, IYY10–IYY11, IYY12–IYY14, IYY14–IYY15, IYY1–IYY2, IYY2–IYY3, and IYY9–IYY5) and 1 within the LMX scale (LMX4–LMX5), identified through an iterative specification search in which the single largest modification index was freed at each step and indices were recalculated before the next pair was selected (see Table S1 in the Supplementary Materials for the modification index and standardized residual covariance associated with each pair). These 10 residual covariances were freed accordingly. Each of these pairs involves items with closely overlapping content or near-synonymous phrasing within the same subscale (e.g., successive items describing similar facets of feeling disconnected from, or lacking company among, coworkers), consistent with method-related shared variance rather than a substantive third dimension. As a result, model fit improved substantially (χ^2^(971) = 2405.45, χ^2^/df = 2.48, CFI = 0.857, TLI = 0.848, RMSEA = 0.065; see Table 2). While the CFI remains below the conventional 0.90 criterion, RMSEA (0.065) falls within the acceptable range. This finding is not uncommon in models with a large number of indicators and degrees of freedom, where CFI is known to be more sensitive to model complexity than RMSEA (Kenny & McCoach, 2003). We therefore treat the measurement model’s fit as marginal rather than fully adequate, rather than relying on any single fit index or a more lenient threshold to characterize it as acceptable. Notably, all factor loadings were above the 0.32 criterion specified by Tabachnick and Fidell (2014) and all item-dimension relationships were found to be significant. Standardized loadings ranged between 0.452 and 0.832 for workplace loneliness items, 0.438 and 0.854 for LMX, 0.393 and 0.776 for contextual performance, and 0.501 and 0.930 for task performance (the complete set of standardized loadings for all items is reported in Table S2 of the Supplementary Materials). The single lowest-loading item (a contextual performance item, λ = 0.393) was retained rather than removed because its loading nonetheless exceeded the minimum criterion of 0.32 recommended by Tabachnick and Fidell (2014), and because its removal would have altered the content coverage of an established, previously validated scale (Motowidlo & Van Scotter, 1994). Moreover, the contextual performance construct’s composite reliability remained strong (CR = 0.913) even with this item included, indicating that its lower loading did not materially compromise the reliability of the latent construct. The second-order loadings of emotional deprivation and social companionship on the general workplace loneliness factor were both strong and significant within the full measurement model as well (λ = 0.848 and 0.986, respectively, *p* < 0.001).

To further examine discriminant validity, the Heterotrait–Monotrait ratio of correlations (HTMT) was calculated for all pairs of constructs, following the procedure recommended by Henseler et al. (2015). All HTMT values remained below the conservative threshold of 0.85 recommended for conceptually distinct constructs: LMX and workplace loneliness (HTMT = 0.443), LMX and task performance (HTMT = 0.104), LMX and contextual performance (HTMT = 0.235), workplace loneliness and task performance (HTMT = 0.367), workplace loneliness and perceived contextual performance (HTMT = 0.400), and task and contextual performance (HTMT = 0.775). Although the HTMT value between task and contextual performance (0.775) was the highest observed, it remained below the recommended cutoff, providing additional support for the discriminant validity of the two performance dimensions despite their relatively high AVE-based overlap. Having established that the four constructs were empirically distinguishable, composite scores were subsequently generated by averaging the corresponding items for LMX, workplace loneliness, task performance, and contextual performance, which were used in the subsequent regression analyses.

The participants’ responses may have been influenced by the Common Method Variance (CMV) problem, as data were collected from participants simultaneously using the same method. Firstly, Harman’s Single-Factor Test was employed to assess whether this problem poses a significant concern in the results. The four factors were obtained in the initial unrotated solution. The first factor explained only 25% of the variance. The factor analysis did not reveal a single factor, nor did a general factor explain the majority of the covariance among the measures. These results suggest that CMV is unlikely to account for the pattern of findings in a substantial way, though the possibility of some inflation in the observed associations cannot be fully excluded given the single-source design. Due to the limitations and criticisms of Harman’s test, common method variance was re-examined using an item-level common latent factor (CLF) approach following the suggestion of Podsakoff et al. (2003). A baseline four-factor CFA model (LMX, workplace loneliness, task performance, contextual performance) was first estimated. A method factor was then added with freely estimated loadings on all 46 observed indicators, its variance fixed to 1 for identification, and its covariances with the four substantive factors constrained to zero. Model fit was compared between the baseline and CLF models, and the standardized trait-factor loadings and method-factor loadings were compared item by item; the squared standardized method-factor loading was interpreted as the percentage of item variance attributable to common method variance (Williams et al., 2010). The significant chi-square difference indicates that CMV could affect the findings to a certain extent (Δχ^2^ (df = 45) = 419.69, *p* < 0.001). At the item level, shared method variance was relatively modest for task and contextual performance items (M = 12.8% and 12.2%, respectively) but moderate for LMX and workplace loneliness items (M = 32.2% and 34.5%, respectively). As a robustness check, composite scores were additionally recalculated as weighted averages using the CMV-adjusted (trait-only) standardized loadings from the CLF model, rather than as simple item means. The direction and statistical significance of the key associations obtained with these CMV-adjusted composites matched those obtained with the original, unadjusted (simple-mean) composite scores used in the primary analyses.

After CMV and CFAs, the correlations between the variables were assessed and found to be generally aligned with the expected direction. Perceived task and perceived contextual performance were significantly and negatively correlated with workplace loneliness (see Table 3). Workplace loneliness was also significantly and negatively correlated with LMX (r = −0.399, *p* < 0.01), indicating that employees reporting higher quality leader–member exchange tended to report lower levels of workplace loneliness. No statistically significant relationship was observed between LMX and perceived task performance. However, a significant and positive relationship existed between LMX and perceived contextual performance.

### 4.2. Results of Hypothesis Testing

The moderating effect of LMX on the relationship between workplace loneliness and perceived job performance was examined using Model 1 of the PROCESS Macro, version 4.2, for SPSS (Hayes, 2018). Separate regression analyses were conducted for perceived task and perceived contextual performance. To rule out multicollinearity, variance inflation factors (VIF) were examined for all predictors in both regression models. The VIF values for the task performance model (WPL: VIF = 1.20; LMX: VIF = 1.19; WPL × LMX: VIF = 1.01) and the contextual performance model (Age: VIF = 1.00; WPL: VIF = 1.21; LMX: VIF = 1.19; WPL × LMX: VIF = 1.01) were between 1.01 and 1.21, which are well below the conventional threshold of 10 (and the more conservative threshold of 5), indicating that multicollinearity was not a threat to the stability or interpretability of the regression estimates.

Prior research suggests that older employees tend to have accumulated greater job-related experience, more established relationships with supervisors, and potentially different socio-emotional priorities at work, all of which could independently shape both task-related and interpersonal (contextual) work behaviors (e.g., Carstensen, 2006; Ng & Feldman, 2008). Based on this theoretical foundation, we controlled for age in the model of task performance and contextual performance. Age was not significantly related to the outcome in the task performance model (b = 0.01, *p* > 0.05), and inclusion of age did not alter the pattern or significance of the main effects. Therefore, consistent with recommendations to retain only theoretically justified controls that also demonstrate an empirical relationship with the outcome (Bernerth & Aguinis, 2016), results are reported without age as a covariate for parsimony. Age was positively and significantly associated with the outcome in the perceived contextual performance model (b = 0.08, *p* < 0.01), which is in line with the theoretical expectation that more experienced employees may perform more contextual/citizenship-oriented behaviors, and was therefore included as a covariate in the reported analysis.

In the first regression analysis, perceived task performance was regressed on the mean-centered scores of LMX, workplace loneliness and their interaction term. As shown in Table 4, although LMX was not significantly associated with task performance, the association between workplace loneliness and perceived task performance was statistically significant. Workplace loneliness was negatively associated with perceived task performance (b = −0.27, SE = 0.04, t = −6.69, *p* < 0.001, 95% CI [−0.34, −0.19]). Thus, H1, which predicted a negative association between workplace loneliness and perceived task performance, was supported. The interaction term between workplace loneliness and LMX was statistically significant in the task-performance model (b = −0.11, SE = 0.04, t = −2.70, *p* = 0.007, 95% CI [−0.20, −0.03]).

**Figure 2:**
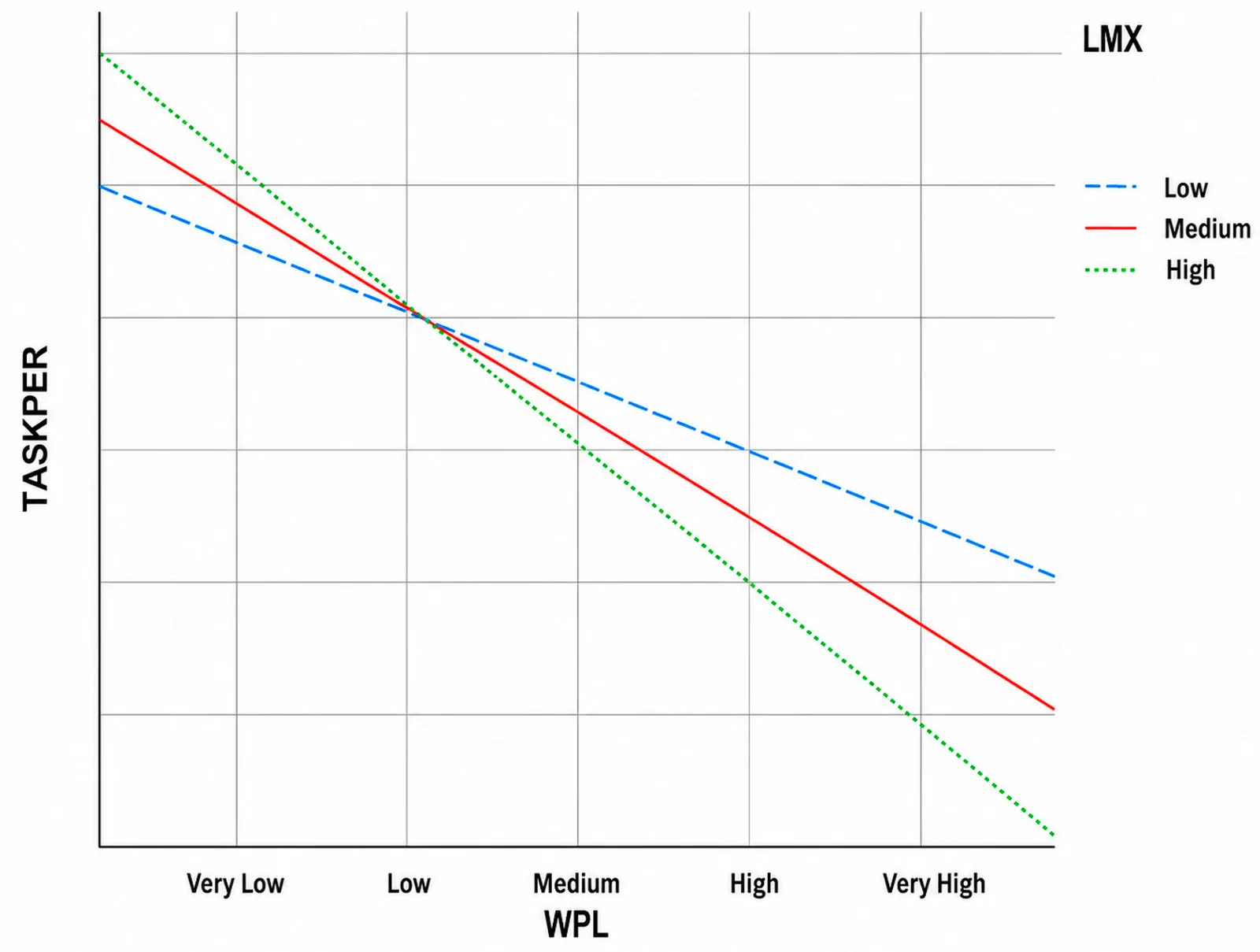
The Moderating Role of LMX in the Association between Workplace Loneliness and Perceived Task Performance.

As shown in Figure 2, workplace loneliness was negatively associated with perceived task performance at each of the three examined levels of LMX. The conditional effect of workplace loneliness was significant when LMX was low (b = −0.18, SE = 0.05, t = −3.66, *p* < 0.001, 95% CI [−0.27, −0.08]), at its mean level (b = −0.27, SE = 0.04, t = −6.69, *p* < 0.001, 95% CI [−0.34, −0.19]), and when LMX was high (b = −0.35, SE = 0.05, t = −6.51, *p* < 0.001, 95% CI [−0.46, −0.25]). The negative association between workplace loneliness and perceived task performance was therefore stronger under high-LMX conditions. In absolute terms, the conditional effect was approximately twice as large at high LMX as at low LMX. Although the interaction effect was statistically significant, its direction was opposite to that proposed in H3. Rather than weakening the negative association between workplace loneliness and perceived task performance, high-quality LMX strengthened it. Therefore, H3 was not supported.

In the second regression analysis (see Table 4), perceived contextual performance was regressed on age, the mean-centered scores of LMX and workplace loneliness, and their interaction term. After controlling for age, the association between workplace loneliness and perceived contextual performance was statistically significant. Workplace loneliness was negatively associated with perceived contextual performance (b = −0.24, SE = 0.04, t = −6.69, *p* < 0.001, 95% CI [−0.31, −0.17]). Thus, H2, which predicted a negative association between workplace loneliness and perceived contextual performance, was supported. As in task performance, the main effect of LMX on contextual performance was not statistically significant. However, the moderating effect of LMX on the relationship between workplace loneliness and perceived contextual performance was found to be significant (b = −0.16, SE = 0.04, t = −4.16, *p* < 0.001, 95% CI [−0.23, −0.08]).

**Figure 3:**
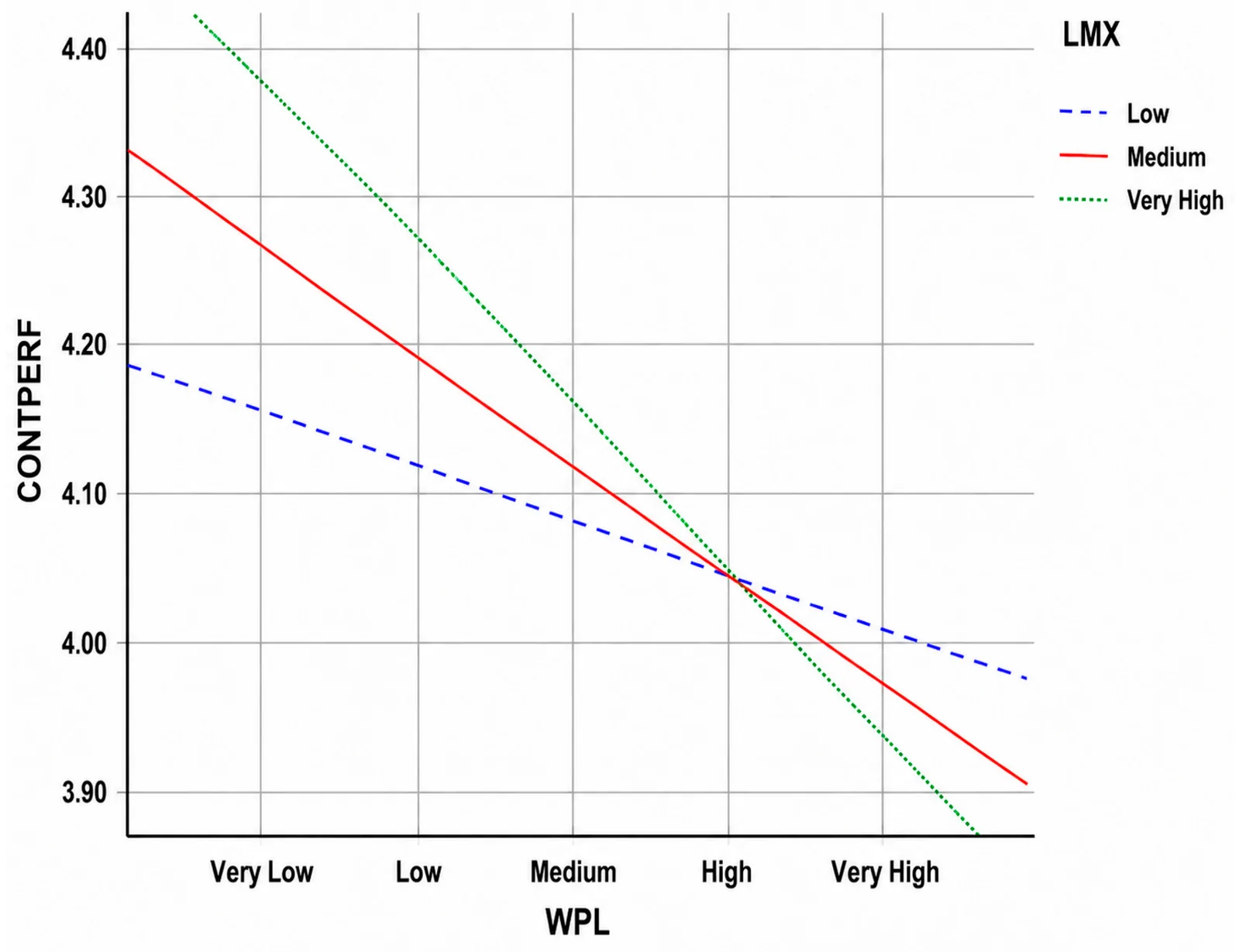
The Moderating Role of LMX in the Association between Workplace Loneliness and Perceived Contextual Performance.

Figure 3 shows that workplace loneliness was negatively associated with perceived contextual performance at each of the three examined levels of LMX. The conditional effect was significant when LMX was low (b = −0.12, SE = 0.04, t = −2.67, *p* = 0.007, 95% CI [−0.21, −0.03]), at its mean level (b = −0.24, SE = 0.04, t = −6.69, *p* < 0.001, 95% CI [−0.31, −0.17]), and when LMX was high (b = −0.37, SE = 0.05, t = −7.40, *p* < 0.001, 95% CI [−0.46, −0.27]). Thus, the negative association between workplace loneliness and contextual performance became progressively stronger as LMX increased. In absolute terms, the conditional effect was approximately three times as large at high LMX as at low LMX. As with perceived task performance, the moderating effect of LMX on perceived contextual performance did not follow the buffering pattern proposed in H4: high-quality LMX amplified, rather than mitigated, the negative association between workplace loneliness and perceived contextual performance. Accordingly, H4 was not supported.

As an additional sensitivity analysis, the principal models were re-estimated using the full sample (*N* = 404) and compared with the results obtained from the final analytic sample (*N* = 352). Overall, the two analyses showed a similar pattern of findings. The negative associations of workplace loneliness with perceived task performance (*N* = 352: b = −0.27, *p* < 0.001; *N* = 404: b = −0.20, *p* < 0.001) and perceived contextual performance (*N* = 352: b = −0.24, *p* < 0.001; *N* = 404: b = −0.17, *p* < 0.001) remained significant in both samples. The workplace loneliness × LMX interaction for contextual performance was also negative and significant in both samples (*N* = 352: b = −0.16, *p* < 0.001; *N* = 404: b = −0.11, *p* = 0.006). For task performance, the interaction coefficient was negative in both samples (*N* = 352: b = −0.11, *p* = 0.007; *N* = 404: b = −0.03, *p* = 0.513), reaching statistical significance only in the final sample. This discrepancy reflects a reduction in the point estimate itself (from b = −0.11 to b = −0.03), not merely a loss of power; because the 52 excluded cases were originally identified as outliers, their inclusion may introduce noise that attenuates this comparatively modest interaction. This pattern is revisited in the Discussion (Section 5) and Limitations (Section 7) sections.

## 5. Discussion

The current study examined the association between workplace loneliness and two aspects of perceived job performance, namely task and contextual performance, as well as the interactive effects of LMX on these relationships. We examined the moderation model using data from 352 white-collar employees working in various organizations. The findings indicate that workplace loneliness is negatively associated with both perceived task and perceived contextual performance. Consistent with the prior research (Ozcelik & Barsade, 2018), loneliness may be associated with the depletion of the social and emotional resources employees need to sustain task engagement. As employees lose access to peer connections and informational exchanges, they may be left with diminished cognitive and motivational reserves, which is a resource deficit that may undermine their capacity to fulfill core job duties (Ozcelik & Barsade, 2018). Furthermore, reduced interaction frequency with co-workers may hinder information exchange, which has been linked to lower perceived task performance.

In addition to task performance, workplace loneliness was also found to be negatively associated with contextual performance. Since extra-role behaviors require discretionary effort and emotional investment beyond formal role demands, they are among the first casualties of resource depletion. When loneliness is associated with a reduced sense of belonging and fewer social resources, the motivational reserves needed to sustain voluntary contributions may also be reduced (Ozcelik & Barsade, 2018). However, loneliness may also be associated with a diminished desire to establish connections with others. This isolation and lack of interaction may weaken employees’ sense of workplace belonging and their connection to the organization. As Ozcelik and Barsade (2018) assert, individuals who experience feelings of loneliness within an organization seem to have a pessimistic perspective on the organizations in which they are employed and believe that their organizations do not sufficiently satisfy their personal needs for socialization and belonging. Thus, these employees may be less inclined to exceed formal job requirements, engage in fewer extra-role behaviors, and consequently report lower levels of contextual performance (Firoz & Chaudhary, 2022; Lam & Lau, 2012).

Regarding the boundary conditions, neither H3 nor H4 was supported. Contrary to expectations, and rather than buffering the adverse associations of workplace loneliness with perceived task and perceived contextual performance, high-quality LMX strengthened both relationships. The additional sensitivity analysis generally supported this pattern: the contextual-performance interaction remained significant in the full sample, while the task-performance interaction retained the same negative direction but did not reach statistical significance in the full-sample analysis. This pattern was not hypothesized a priori; the explanations considered below were therefore developed post hoc and, because the underlying psychological mechanisms were not directly measured, should be interpreted as tentative interpretations rather than as empirically established or confirmatory mechanisms. One possible post hoc interpretation of this finding may be derived from the betrayal framework (Restubog et al., 2010). Within this framework, perceived betrayal may arise when a trusted party’s action or failure to act is experienced as a serious breach of relational expectations (Restubog et al., 2010). From this perspective, it is possible that lonely employees with high-quality LMX may experience greater disappointment when their expectations of supervisory support are not met. An employee who feels lonely but has a good relationship with their leader may develop high expectations of support from that leader. If those expectations are not met, the employee may feel disappointed, which could reinforce feelings of loneliness and lead to increased sensitivity to adverse work conditions. Such disappointment could potentially intensify the negative association between workplace loneliness and perceived performance. This finding is consistent with previous studies reporting the dark side of LMX. In particular, Anand and Mishra (2021) have demonstrated that the positive relationship between loneliness and emotional exhaustion is more pronounced for employees with high-quality LMX. A similar pattern was reported by Restubog et al. (2010), who found that psychological contract breach was more strongly associated with lower organizational citizenship behavior and in-role performance under high-quality LMX. In line with the betrayal framework, employees in high-quality relationships may hold stronger expectations of support, and unmet expectations may be associated with lower in-role performance and extra-role behavior.

A second plausible interpretation of the observed interaction pattern concerns performance pressure. High-quality exchange partnerships are characterized not just by “support and resource provision” but also by heightened role expectations. Indeed, managers who develop an employee through mentorship, tough assignments, and resource sharing also concurrently convey that good performance is anticipated in return (Law et al., 2000; Wu, 2018). These increased performance demands may constitute an additional burden for employees who are already socially depleted by workplace loneliness. Rather than buffering the negative association between loneliness and performance, the heightened expectations associated with a strong LMX relationship may therefore intensify this association. This performance-pressure explanation differs from the betrayal concept in that it involves no perceived violation of trust, but rather the amplification of performance pressure from the mismatch between relational demands and exhausted social resources.

The last possible interpretation is that even though the employee has high-quality LMX relationships with their leader, they may still feel lonely. When an employee feels lonely and relies heavily on their leader for connection, they may become more sensitive to any perceived imbalance in their relationships with co-workers. Researchers have, in fact, framed loneliness as a regulatory cycle that individuals find difficult to break (Ozcelik & Barsade, 2018). Prolonged loneliness can lead to the development of negative thought patterns, which in turn hinder social connections (Ozcelik & Barsade, 2018) and impair expected performance levels. Furthermore, employees with high-quality relationships with their supervisors may exhibit diminished motivation for creating new connections and make more use of their leaders’ social networks. Taken as a whole, the betrayal framework and other mechanisms discussed above could provide reasonable interpretations of the present findings, although these interpretations should be considered tentative given the cross-sectional design and the fact that the proposed mechanisms were not directly assessed.

These findings can also be interpreted through a cultural lens. In collectivist cultures, individuals often feel a strong attachment to their organizational members (Luksyte et al., 2022) and attach utmost value and importance to the development and preservation of positive interpersonal relationships (Kağıtçıbaşı, 1996). Thus, in a relatively collectivist culture (Hofstede, 1980), Turkish employees could be sensitive to workplace loneliness and thereby give reactions that are more negative. As cultural dimensions were not directly tested in the study, these cultural characteristics are provided as contextual background for interpreting the present findings rather than as empirically established explanations. It is still debatable whether collectivism and power distance moderate the loneliness–performance association as proposed here. Therefore, future cross-cultural research should tackle this question by directly measuring cultural values.

## 6. Practical Implications

Our study provides valuable insights into managing workplace loneliness. More broadly, drawing on the wider literature on workplace loneliness interventions, we offer the following general recommendations. Considering the negative associations of workplace loneliness with perceived job performance dimensions, managers or practitioners should aim to reduce or eliminate feelings of loneliness. We recommend managers create an organizational culture that supports increased employee interaction, implement mentoring systems for support and guidance, and actively seek employees’ opinions, experiences, and emotions (Sullivan & Bendell, 2023; Y. Yang et al., 2024). In addition, organizational structure could be redesigned to allow individuals from different departments to establish new interactions and share their opinions. For example, forming team-based structures may enable employees to discuss their opinions and feel connected to the members of other departments.

In addition to organizational interventions, managers could provide employees with special training programs or workshops to enhance their emotional intelligence and communication skills. Such personalized interventions would better equip employees to form social connections (Sullivan & Bendell, 2023). Given the fact that perception of loneliness could occur even in the presence of others, managers could design special programs and integrate mindfulness-oriented approaches to increase the resilience of employees to deal with negative feelings arising from loneliness (Sullivan & Bendell, 2023; Xin et al., 2024). We believe that employees do not necessarily need high-quality relationships with specific people at work to feel a sense of belonging. Instead, they can find purpose through their work itself to prevent workplace loneliness.

Directly building on our findings, this study revealed an unexpected, intensifying rather than buffering pattern associated with high-quality LMX, challenging the common assumption that having high-quality relations with supervisors could provide employees with necessary resources for enhanced job performance and protection against loneliness and isolation. The results suggest that the negative association between loneliness and performance may not be fully addressed through high-quality LMX alone. Managers should therefore avoid assuming that employees with whom they have high-quality relationships are well supported and free from struggles with their co-workers. As noted by Sherony and Green (2002), differences in the quality of coworkers’ LMX relationships with the same leader may influence the quality of coworker-exchange relationships. Such differences could potentially weaken broader peer relationships and contribute to feelings of loneliness. Thus, managers need to provide more active emotional support and look beyond surface-level interactions with the employees (Ozcelik & Barsade, 2018). They are suggested to develop a deeper understanding of the interpersonal problems among employees and act as intermediaries or liaisons to solve those problems.

In addition, managers should be aware that their high-quality relationships with employees might induce additional tension for employees experiencing isolation (Anand & Mishra, 2021; Gerstner & Day, 1997; Wu, 2018). Employees might feel pressure to maintain good relations with their managers by performing at a high level, which could be especially challenging for those experiencing isolation and a lack of co-worker support. Thus, managers are encouraged to balance task demands with emotional and social support to ensure employees do not feel additional tension.

Rather than relying on individual leader–member interactions to mitigate loneliness, leaders and organizations should invest in developing a holistic, inclusive environment that directly addresses the issue. Furthermore, organizations could establish broader social support networks, such as team-building activities, to ensure that isolated, lonesome employees have a variety of social connections beyond their immediate supervisors.

## 7. Limitations and Future Research Directions

This research is subject to several limitations due to its methodology and research scope. Firstly, although data on participants’ employment sector were collected, information identifying the specific organization to which each participant belonged was not obtained. In addition, information on participants’ industries and organizational size was not collected, which limits the specificity with which the organizational context of the sample can be characterized. As a result, it was not possible to account for potential non-independence of observations arising from employees being nested within the same organization (e.g., via cluster-robust standard errors). Future research collecting organization-level identifiers could examine whether the observed associations vary systematically across organizational contexts.

Secondly, the study relies on employees’ assessments of their own performance. We assume that employees’ evaluations are relatively accurate indicators of performance (Heidemeier & Moser, 2009); however, we cannot rule out the possibility that perceptual and evaluative processes could contribute to the findings in ways that are unrelated to the relationship between loneliness and performance. The mean of self-reported task performance in the present sample was also relatively high (M = 4.45, SD = 0.45), suggesting a potential ceiling effect and restricted variance in this variable. Future studies could supplement self-rated task performance with supervisor or peer ratings to obtain a wider range of scores.

Thirdly, this study does not consider other individual (e.g., ability, motivation, personality) and organizational factors (e.g., availability of resources, organizational structure, and culture, etc.) hindering or enhancing performance. Future research could integrate the aforementioned factors into their research model to offer a deeper insight into the relationship among loneliness, performance, and LMX.

Fourthly, the cross-sectional nature of this study did not allow a causal inference to be made regarding associations between workplace loneliness and perceived task and perceived contextual performance. Accordingly, reverse or reciprocal relationships cannot be ruled out; for example, lower perceived performance may precede or contribute to greater perceptions of workplace loneliness, rather than the association necessarily operating only from workplace loneliness to perceived performance. Thus, the observed findings should be interpreted as cross-sectional associations rather than as evidence of causal changes in objective performance. Future researchers could employ time-series-based experimental studies to determine causality and gain a better understanding of the evolving nature of these relationships. Furthermore, utilizing the self-report questionnaires might have resulted in CMV bias. All focal dimensions (workplace loneliness, LMX, task performance, and contextual performance) were measured at a single time point from the same respondents using the same survey instrument. Such a cross-sectional design characteristic is known to inflate observed correlations and complicate causal inference. Although Harman’s single-factor test did not indicate that a single general factor accounted for the majority of covariance among the study variables, the CLF analysis revealed a low-to-moderate level of shared method variance. This suggests that associations involving workplace loneliness in the present study, including its interaction with LMX, may be inflated to some degree by shared method variance beyond what conventional CMV diagnostics detect, and should be interpreted with corresponding caution. Therefore, future research is recommended to apply multi-source designs, e.g., collecting loneliness and LMX evaluations from employees and receiving performance ratings from supervisors or peers, or make use of experience-sampling techniques allowing for a more rigorous control of CMV.

The use of non-probability snowball sampling may have limited the representativeness of the sample and, consequently, the generalizability of the findings. Future research could employ probability-based or more diverse sampling strategies to examine the robustness of the findings across broader employee populations. Relatedly, a supplementary multivariate outlier check based on Mahalanobis distance flagged 19 cases in the final analytic sample that were not identified by the univariate screening criterion. These cases were retained because they were distinguished primarily by markedly low LMX scores rather than by anomalous responding, and sensitivity analyses confirmed that excluding them did not alter the direction or significance of the key interaction effects; nonetheless, future research should verify the robustness of the present findings in independent, more heterogeneous samples. Separately, the full-sample sensitivity analysis reported in the Results section (*N* = 404, including the 52 cases excluded via univariate screening) suggested that the task-performance interaction may be somewhat more sensitive to sample specification than the contextual-performance interaction, as it retained the same direction but did not reach statistical significance in that full-sample analysis. Future studies may further examine the stability of this finding across independent samples. Given that H3 and H4 were not supported and that high-quality LMX unexpectedly strengthened, rather than weakened, the negative associations between workplace loneliness and performance, future research should replicate this pattern across diverse organizational settings and directly test, using a priori hypotheses and measures collected prior to data analysis, the post hoc mechanisms proposed here (e.g., betrayal, performance pressure) before firm conclusions are drawn regarding this boundary condition.

## 8. Conclusions

The current literature has largely emphasized the beneficial associations of high-quality LMX with employee outcomes. However, H3 and H4 were not supported. Nonetheless, the data indicated that the negative association between loneliness and both perceived task and perceived contextual performance was stronger when employees reported high-quality relations with their supervisors. Moreover, the study enriches our understanding of the relationship between loneliness and performance by extending it to less studied cultural contexts. These insights highlight the importance of interpersonal relationships by showing the negative association of loneliness with performance and the plausible dark side of high-quality LMX. Consequently, managers should recognize that high-quality LMX may not always function as a protective relational resource and should remain attentive to employees’ broader interpersonal experiences at work.

## Figures and Tables

**Table 1:** Demographic characteristics of participants.

	Frequency (n)	Percentage (%)
Gender		
Female	184	52.3
Male	168	47.7
Age		
18–26	47	13.4
27–35	53	15.1
36–44	93	26.4
45 years and above	159	45.2
Educational Status		
High School	19	5.4
Bachelor’s degree	240	68.2
Master’s degree	79	22.4
Ph.D.	14	4.0
Sector		
Public Sector	224	63.6
Private Sector	128	36.4

**Table 2:** Goodness-of-Fit Statistics of the Measurement Model.

	Original Model	Revised Model
CMIN	2647.32	2405.45
CFI	0.834	0.857
RMSEA	0.070	0.065
χ^2^/df	2.70 (df = 981)	2.48 (df = 971)
TLI	0.823	0.848
SRMR	0.081	0.079

Note. CMIN = chi-square; CFI = comparative fit index; RMSEA = root mean square error of approximation; χ^2^/df = chi-square/degrees of freedom; TLI = Tucker–Lewis index; SRMR = standardized root mean square residual.

**Table 3:** Bivariate correlations and descriptive statistics.

Variables	Mean	SD	1	2	3	4	5	6	7	8
1. Gender			1							
2. Age			0.356 **	1						
3. Education			−0.002	−0.150 **	1					
4. Sector			−0.155 **	−0.453 **	0.069	1				
5. WPL	2.10	0.63	0.111 *	0.049	0.133 *	0.040	1			
6. PTaskPer	4.45	0.45	−0.097	0.039	0.048	−0.025	−0.332 **	1		
7. PContPer	4.16	0.43	0.011	0.167 **	−0.025	−0.032	−0.357 **	0.685 **	1	
8. LMX	3.36	0.78	0.054	0.069	0.114	0.075	−0.399 **	0.095	0.219 **	1

Notes. * *p* < 0.05, ** *p* < 0.01; Gender (1 = Female, 2 = Male); Age (1 = 18–26, 2 = 27–35, 3 = 36–44, 4 = 45 years and above); Educational Status (1 = High School, 2 = Bachelor’s degree, 3 = Master’s degree, 4 = PhD); Sector (1 = Public Sector, 2 = Private Sector). WPL = workplace loneliness; PTaskPer = perceived task performance; PContPer = perceived contextual performance; LMX = leader–member exchange.

**Table 4:** Regression results for moderation.

Dependent Variable: Perceived Task Performance F(3, 348) = 17.35, *p* < 0.001, R^2^ = 0.13						
Predictor	b	SE	T	*p*	LLCI	ULCI
Constant	4.43	0.024	184.26	*p* < 0.001	4.38	4.48
WPL	−0.27	0.04	−6.69	*p* < 0.001	−0.34	−0.19
LMX	−0.03	0.03	−0.93	0.35	−0.09	0.03
WPL × LMX	−0.11	0.04	−2.70	0.007	−0.20	−0.03
Conditional effect						
LMX	Effect	SE	T	*p*	LLCI	ULCI
−0.7809 (−1 SD)	−0.18	0.05	−3.66	0.0003	−0.27	−0.08
0.0000 (mean)	−0.27	0.04	−6.69	*p* < 0.001	−0.34	−0.19
0.7809 (+1 SD)	−0.35	0.05	−6.51	*p* < 0.001	−0.46	−0.25
Dependent Variable: Perceived Contextual Performance F(4, 347) = 22.93, *p* < 0.001, R^2^ = 0.21						
Predictor	b	SE	T	*p*	LLCI	ULCI
Constant	3.90	0.06	62.09	*p* < 0.001	3.77	4.02
Age	0.08	0.02	3.87	*p* < 0.001	0.037	0.11
WPL	−0.24	0.04	−6.69	*p* < 0.001	−0.31	−0.17
LMX	0.05	0.03	1.75	0.08	−0.006	0.11
WPL × LMX	−0.16	0.04	−4.16	*p* < 0.001	−0.23	−0.08
Conditional effect						
LMX	Effect	SE	T	*p*	LLCI	ULCI
−0.7809 (−1 SD)	−0.12	0.04	−2.67	0.007	−0.21	−0.03
0.0000 (mean)	−0.24	0.04	−6.69	*p* < 0.001	−0.31	−0.17
0.7809 (+1 SD)	−0.37	0.05	−7.40	*p* < 0.001	−0.46	−0.27

Notes: b: Unstandardized regression estimate, SE: Standard error of unstandardized estimate, LLCI: confidence interval lower bound, ULCI: confidence interval upper bound, WPL: Workplace Loneliness, LMX: Leader–Member Exchange.

## Data Availability

The data presented in this study are available from the corresponding author upon reasonable request. The data are not publicly available due to participant privacy and ethical considerations.

## Supplementary Materials

The following supporting information can be downloaded at https://www.mdpi.com/article/10.3390/bs16091608/s1, Table S1: Modification indices and standardized residual covariances for the ten residual covariance pairs freed in the revised measurement model; Table S2: Complete standardized factor loadings for the revised measurement model.

## Abbreviations

The following abbreviations are used in this manuscript:

AVE	Average Variance Extracted
CFA	Confirmatory Factor Analysis
CFI	Comparative Fit Index
CMV	Common Method Variance
COR	Conservation of Resources
CR	Composite Reliability
LMX	Leader–Member Exchange
RMSEA	Root Mean Square Error of Approximation
WPL	Workplace Loneliness
