# Supplementary material for: Lonely at Work: The Moderating Role of Leader–Member Exchange in the Association Between Workplace Loneliness and Perceived Task and Contextual Performance
Source: Behav Sci (Basel). 2026 Sep 9;16(9):1608. doi: 10.3390/bs16091608 (PMC13603593; doi:10.3390/bs16091608)
Supplement: Supplementary file 1 [file behavsci-16-01608-s001.zip › behavsci-4533520-supplementary.pdf]

behavioral sciences

MDPI

Supplementary Materials

Table S1. Modification Indices and Standardized Residual Covariances for the Ten Residual Covariance Pairs Freed in the Revised Measurement Model.

Item Pair	Scale	Modification Index ($\Delta\chi^2$, 1 df)	Standardized Residual Covariance	Step Freed
IYY3–IYY4	Workplace Loneliness (ED)	68.29	.211	1
IYY5–IYY6	Workplace Loneliness (SC)	42.89	.151	2
IYY2–IYY9	Workplace Loneliness (ED)	29.01	-.094	3
IYY10–IYY11	Workplace Loneliness (SC)	23.82	.115	4
IYY12–IYY14	Workplace Loneliness (SC)	21.42	.085	5
IYY14–IYY15	Workplace Loneliness (SC)	18.48	.052	6
IYY1–IYY2	Workplace Loneliness (ED)	16.14	.089	7
IYY2–IYY3	Workplace Loneliness (ED)	16.33	.122	8
IYY9–IYY5	Workplace Loneliness (ED–SC)	14.50	.118	9
LMX4–LMX5	Leader-Member Exchange	38.44	.197	10

Note. ED = Emotional Deprivation subscale; SC = Social Companionship subscale of the Workplace Loneliness Scale (Wright et al., 2006). Modification indices and standardized residual covariances were obtained through an iterative specification search: at each step, the single largest modification index among the remaining candidate item pairs sharing similar wording within the same subscale was identified and freed, after which the model was re-estimated and modification indices were recalculated before the next pair was selected (steps 1–9 within the Workplace Loneliness scale; step 10 within the LMX scale). All ten pairs involve items with closely overlapping content or near-synonymous phrasing, consistent with method-related shared variance rather than a substantive additional dimension. Modification indices approximate a likelihood-ratio ($\Delta\chi^2$, 1 df) test for the residual covariance parameter; a value above 3.84 corresponds to $p < .05$.

Table S2. Complete Standardized Factor Loadings for the Revised Measurement Model.

Item	Construct/Subscale	Standardized Loading
IYY1	Emotional Deprivation (Workplace Loneliness)	0.607
IYY2	Emotional Deprivation (Workplace Loneliness)	0.791
IYY3	Emotional Deprivation (Workplace Loneliness)	0.527
IYY4	Emotional Deprivation (Workplace Loneliness)	0.708
IYY7	Emotional Deprivation (Workplace Loneliness)	0.766
IYY8	Emotional Deprivation (Workplace Loneliness)	0.842
IYY9	Emotional Deprivation (Workplace Loneliness)	0.754
IYY13	Emotional Deprivation (Workplace Loneliness)	0.486
IYY5	Social Companionship (Workplace Loneliness)	0.738
IYY6	Social Companionship (Workplace Loneliness)	0.680
IYY10	Social Companionship (Workplace Loneliness)	0.759
IYY11	Social Companionship (Workplace Loneliness)	0.635
IYY12	Social Companionship (Workplace Loneliness)	0.761
IYY14	Social Companionship (Workplace Loneliness)	0.707
IYY15	Social Companionship (Workplace Loneliness)	0.853
IYY16	Social Companionship (Workplace Loneliness)	0.757
LMX1	Leader-Member Exchange	0.641
LMX2	Leader-Member Exchange	0.826
LMX3	Leader-Member Exchange	0.785
LMX4	Leader-Member Exchange	0.438

LMX5	Leader-Member Exchange	0.665
LMX6	Leader-Member Exchange	0.718
LMX7	Leader-Member Exchange	0.854
PGP1	Task Performance	0.861
PGP2	Task Performance	0.930
PGP3	Task Performance	0.915
PGP4	Task Performance	0.755
PGP5	Task Performance	0.501
PGP6	Task Performance	0.746
PGP7	Task Performance	0.760
PBP1	Contextual Performance	0.695
PBP2	Contextual Performance	0.684
PBP3	Contextual Performance	0.683
PBP4	Contextual Performance	0.717
PBP5	Contextual Performance	0.479
PBP6	Contextual Performance	0.484
PBP7	Contextual Performance	0.538
PBP8	Contextual Performance	0.640
PBP9	Contextual Performance	0.753
PBP10	Contextual Performance	0.393
PBP11	Contextual Performance	0.775
PBP12	Contextual Performance	0.719
PBP13	Contextual Performance	0.570
PBP14	Contextual Performance	0.681
PBP15	Contextual Performance	0.550
PBP16	Contextual Performance	0.652

Note. Standardized loadings are from the revised four-factor measurement model (LMX, workplace loneliness [second-order: Emotional Deprivation and Social Companionship], task performance, contextual performance), N = 352, with the ten residual covariances listed in Table S1 freed. All loadings exceed the .32 minimum criterion recommended by Tabachnick and Fidell (2014). The second-order loadings of Emotional Deprivation and Social Companionship on the general Workplace Loneliness factor were $\lambda = .848$ and .986, respectively (both $p < .001$).

References

(Wright et al., 2006) Wright, S. L., Burt, C. D., & Strongman, K. T. (2006). Loneliness in the workplace: Construct definition and scale development. *New Zealand Journal of Psychology*, *35*(2), 59–68.

(Tabachnick & Fidell, 2014) Tabachnick, B. G., & Fidell, L. S. (2014). *Using multivariate statistics* (6th ed.). Pearson Education Limited.
